# Heparin-free veno-venous ECMO for airway obstruction: A case report and review of literature

**DOI:** 10.1097/MD.0000000000041098

**Published:** 2024-12-27

**Authors:** Bin Sun, Meiyan Zhou, Rongguo Wang, Qian Liu, Li Yan, Yan Zhang, Jinghao Zhang, Liwei Wang

**Affiliations:** a Department of Anesthesiology, Xuzhou Clinical School of Xuzhou Medical University, Xuzhou Central Hospital, Xuzhou, Jiangsu, China; b Department of Respiratory Medicine, Xuzhou Central Hospital, Xuzhou, Jiangsu, China.

**Keywords:** airway obstruction, case report, heparin-free VV-ECMO, surgical resection, synovial sarcoma

## Abstract

**Rationale:**

Life-threatening airway obstructions caused by tumors demand prompt and effective intervention. Traditional surgical methods are often complicated by bleeding risks, especially with the use of anticoagulation during extracorporeal membrane oxygenation (ECMO). This report investigates the innovative application of heparin-free veno-venous ECMO (VV-ECMO) to minimize bleeding risks while maintaining effective oxygenation during airway obstruction surgeries, thereby offering a safer alternative in high-risk scenarios.

**Patient concerns:**

A 44-year-old female with a history of recurrent synovial sarcoma presented with severe dyspnea, requiring a forced lateral position to breathe. A chest CT scan revealed a complete obstruction of the left mainstem bronchus by a tumor.

**Diagnoses:**

The patient was diagnosed with airway obstruction secondary to metastatic synovial sarcoma.

**Interventions:**

Preemptive heparin-free VV-ECMO was initiated before general anesthesia to maintain oxygenation. Surgical resection of the obstructing tumor was performed using rigid bronchoscopy and high-frequency electrocautery, followed by argon plasma coagulation to control bleeding.

**Outcomes:**

Postoperatively, the patient showed significant improvement in respiratory status. VV-ECMO was successfully weaned off, and the patient was extubated shortly after surgery. She was discharged in stable condition 4 days later.

**Lessons:**

Heparin-free VV-ECMO is an effective strategy for managing airway obstructions in patients at high-risk of bleeding. This case supports the use of ECMO without anticoagulation in airway surgeries, offering a balance between maintaining oxygenation and reducing bleeding complications.

## 1. Introduction

Lung cancer, airway tumor, anterior mediastinal tumor, and other conditions frequently invade or compress the trachea and bronchi. This invasion or compression can lead to severe airway narrowing and, in extreme cases, direct asphyxiation resulting in death. In response to this, bronchoscopic intervention has emerged as the preferred treatment method for the timely and effective relief of various airway obstructions.^[1]^ However, in patients with severe central airway obstruction, the high-risk of severe hypoxemia, respiratory and cardiac arrest often limits this form of treatment. Addressing this issue, research has emphasized that preemptive awake veno-venous extracorporeal membrane oxygenation (VV-ECMO) should be considered as a strategic solution to avoid intraoperative hypoxemia.^[2]^

The use of heparin-free ECMO is particularly significant in reducing the risk of intraoperative bleeding, a common complication associated with anticoagulation during ECMO. Studies have demonstrated that VV-ECMO can be safely performed without systemic anticoagulation, thus minimizing bleeding risks during airway surgeries.^[3]^ This approach is especially beneficial for patients undergoing tumor resection, where bleeding control is paramount. The present case highlights the successful use of heparin-free VV-ECMO in a patient with severe airway obstruction, demonstrating its effectiveness in maintaining adequate gas exchange while reducing intraoperative bleeding risks. This case contributes to the growing body of evidence supporting the safety and efficacy of heparin-free ECMO in critical airway management.^[4–6]^

## 2. Case report

**Figure 1.**
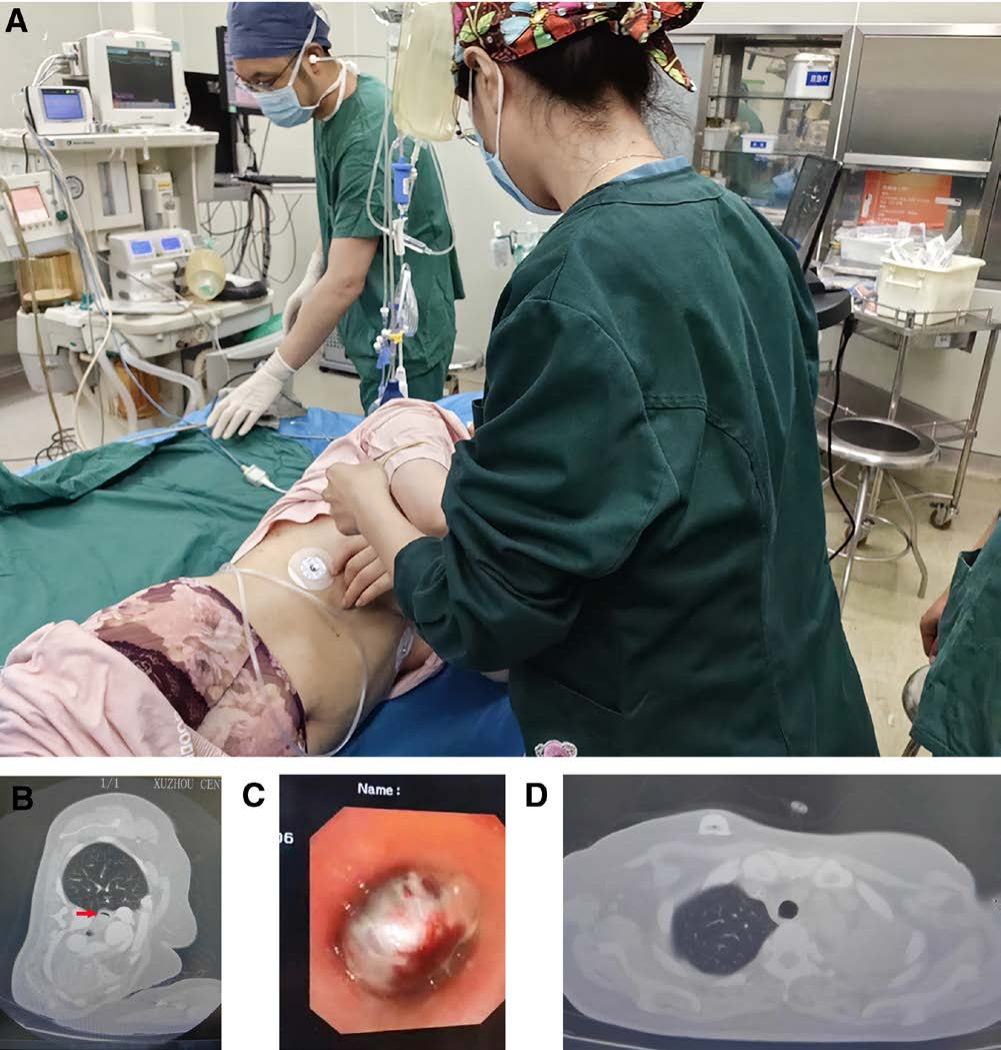
Patient’s position in the operating room and preoperative information. (A) The patient is positioned in the forced left lateral decubitus position in the operating room, prepared for the initiation of heparin-free veno-venous extracorporeal membrane oxygenation (VV-ECMO). (B) Preoperative chest computed tomography (CT) scan showing significant airway obstruction (indicated by the red arrow) due to a mass lesion. (C) Bronchoscopic view of the obstructing mass in the main airway, revealing the extent of the blockage and the need for immediate intervention. (D) Another angle of the preoperative chest CT scan, further illustrating the location and size of the obstructing mass. CT = computed tomography, VV-ECMO = veno-venous extracorporeal membrane oxygenation.

A 44-year-old female presented with severe dyspnea and wheezing, requiring a forced left lateral position to maintain adequate respiration (Fig. 1A). Her medical history included a left axillary synovial sarcoma resected 8 years prior, followed by postoperative recurrence and metastasis to the lung. The patient had undergone multiple unsuccessful treatments, including chemotherapy and comprehensive therapy. A chest computed tomography (CT) scan revealed that the metastasized tumor had invaded the distal trachea, carina, and completely obstructed the left mainstem bronchus (Fig. 1B–D). On admission, she was confined to the left lateral position to maintain airflow through a narrow gap due to gravitational factors, and required supplemental oxygen.

Upon admission, a multidisciplinary consultation was conducted. The experts recommended preemptive initiation of VV-ECMO before the induction of general anesthesia to avoid the risks associated with complete tracheal occlusion. In the operating room, the patient was positioned in the left lateral decubitus position. Hemodynamic monitoring was established with a 20 G cannula in the left radial artery. Preoperative measurements showed a sinus rhythm with a heart rate of 112 bpm, arterial blood pressure of 180/110 mm Hg, and an oxygen saturation of 97% on 4L/min nasal oxygen. Sedation was achieved using low-dose dexmedetomidine infusions, and local anesthesia was administered with lidocaine. A F20 drainage cannula was inserted into the left femoral vein, and a F16 return cannula was positioned in the right internal jugular vein under ultrasound guidance. VV-ECMO was initiated without anticoagulation, maintaining the patient’s oxygen saturation above 98%.

**Figure 2.**
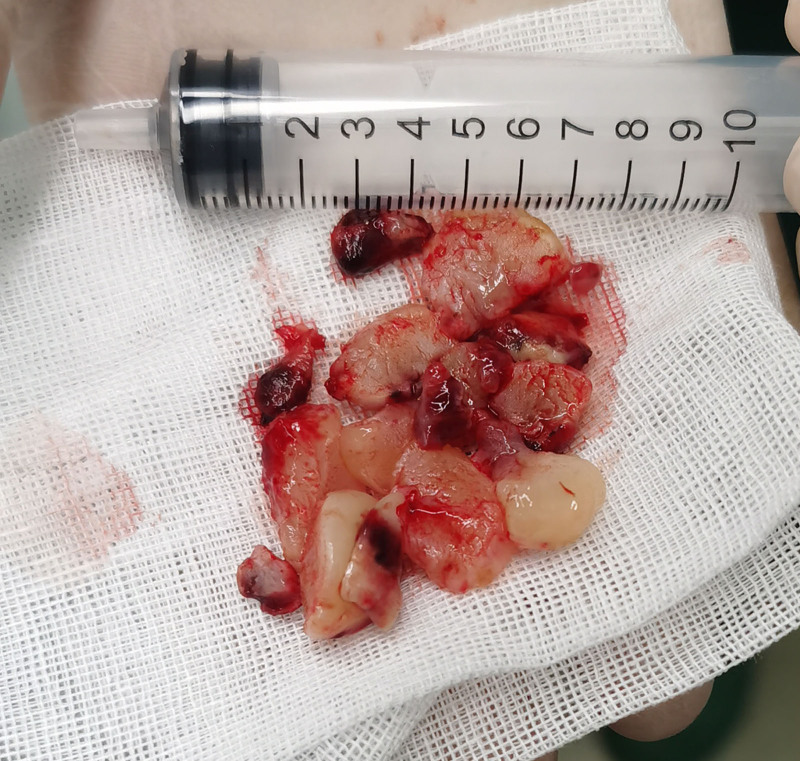
Surgical resection of tumor tissue.

Anesthesia induction was performed with intravenous propofol, cisatracurium, and sufentanil, and maintained with propofol and remifentanil infusions. The patient was repositioned to the supine position, and rigid bronchoscopy revealed a complete central airway obstruction. High-frequency electrocautery was used to remove the airway tumor in the distal trachea, followed by argon plasma coagulation to treat the base. The intraluminal tumor in the left main bronchus was also excised (Fig. 2), clearing the central airway and left main bronchus. Following adequate local hemostasis and suctioning, a 7F endotracheal tube was inserted, and a slow lung recruitment strategy was implemented. Postoperatively, the patient’s vital signs remained stable, and blood gas analysis was normal. ECMO was weaned off successfully.

The patient was extubated 2 hours after transfer to the ICU and moved to a general ward 3 hours later. Her respiratory distress symptoms significantly improved, allowing free position changes with stable vital signs and oxygen saturation. Pathology confirmed the diagnosis of synovial sarcoma. She was discharged on the fourth day post-surgery, with a notable improvement in her condition. Written informed consent was obtained from the patient for the publication of the details of this case report.

## 3. Literature review

We conducted a search in the PubMed database from 2015 to 2024 to identify case reports on heparin-free ECMO. The search focused on English-language publications, with abstracts screened for relevance. Full-text articles in other languages were included if an English abstract was available. The review criteria included patient demographics, clinical presentation, diagnosis, ECMO treatment details, and outcomes. A total of 12 case reports met these criteria and are detailed in Table 1.^[4–15]^

**Table 1:** Summary of cases related to heparin-free ECMO.

Author	Year	Age	Gender	Diagnosis	Type of ECMO	ECMO duration	Heparin	Heparin usage time	Unused heparin time	Number of oxygenator or circuit replacements	Outcome
Araki H^[7]^	2024	39 yr	Female	Amniotic fluid embolism	VA-ECMO	5 d	No	0	5 d	0	Survived
Matsumoto H^[8]^	2023	48 yr	Male	COVID-19 ARDS with refractory oronasal bleeding	VV-ECMO	48 d	Yes (heparin)	19 d	29 d	3 times	Survived
Zhao J^[6]^	2023	58 yr	Male	trachea stenosis with tumor metastasis	VV-ECMO	1 d	No	0	1 d	0	Survived
Durila M^[9]^	2023	43 yr	Male	Respiratory and cardiac failure	VV-ECMO and VA-ECMO	94 d	Yes (enoxaparin)	94 d	0	10 times	Survived
Qiao G^[10]^	2021	46 yr	Male	Respiratory failure due to trauma	VV-ECMO	20 d	No	0	20 d	0	died
Zhong H^[11]^	2020	27 yr	Female	Severe ARDS and cardiac arrest	VA-ECMO	42 d	Yes (heparin)	11 d	31 d	3 times	Survived
Lee YY^[12]^	2020	17 yr	Female	Multiple trauma with traumatic brain injury	VV-ECMO	3 d	No	0	3 d	0	Survived
Ogawa F^[13]^	2019	32 yr	Male	Severe blunt trauma with massive hemothorax	VV-ECMO	10 d	Yes (heparin)	5 d	5 d	0	Survived
Galvagno SM^[14]^	2019	52 yr	Female	Diffuse alveolar hemorrhage and ARDS	VV-ECMO	210 d	Yes (heparin)	80 d	130 d	6 times	Survived
Faulkner AL^[15]^	2019	44 yr	Female	ARDS with aneurysmal subarachnoid hemorrhage	VV-ECMO	9 d	No	0	9 d	0	Survived
Ryu KM^[4]^	2018	48 yr	Male	Pulmonary contusions and bronchial disruption	VV-ECMO	2.5 d	No	0	2.5 d	0	Survived
Wen PH^[5]^	2015	19 yr	Male	Pulmonary contusion and grade IV liver laceration	VV-ECMO	5 d	No	0	5 d	0	Survived

ARDS = acute respiratory distress syndrome, ECMO = extracorporeal membrane oxygenation, VA-ECMO = venoarterial extracorporeal membrane oxygenation, VV-ECMO = veno-venous extracorporeal membrane oxygenation.

## 4. Discussion

In this case, a 44-year-old female with a history of recurrent synovial sarcoma presented with severe dyspnea due to a complete obstruction of the left mainstem bronchus. Following the initiation of heparin-free VV-ECMO, her oxygen saturation was maintained above 98% throughout the procedure. Postoperatively, she demonstrated stable vital signs and normal blood gas analysis. VV-ECMO was successfully weaned off, with no complications related to thrombotic events or bleeding. The patient was transferred to the ICU for further monitoring to prevent airway bleeding, and was extubated 2 hours later. She was moved to a general ward 3 hours after extubation, with continued stable oxygen saturation and the ability to change positions freely. She was discharged in stable condition 4 days post-surgery. Notably, this patient represents the first known case of ECMO implantation performed in the lateral position, a factor that significantly increased the procedural complexity. The lateral positioning posed substantial challenges, particularly in achieving vascular access; however, the use of ultrasound-guided vascular puncture effectively mitigated these difficulties, enabling safe cannula insertion. This case underscores ECMO’s critical role as a bridge to definitive airway management,^[16]^ contributing to the expanding body of evidence supporting its use in managing airway obstructions.

The use of anticoagulation in ECMO therapy presents significant challenges due to the dual risks of bleeding and thrombosis. Heparin, commonly used to prevent thrombotic complications, can lead to severe bleeding, particularly in patients with trauma or those undergoing surgical procedures. Hemorrhagic complications may increase the complexity of surgery, leading to higher incidence rates and mortality, making it essential to carefully balance thrombosis prevention with bleeding risk management.^[14,17]^ The development of heparin-free ECMO protocols has been driven by these concerns. Heparin-free ECMO has been successfully used in trauma patients, those with pulmonary hemorrhage, and in cases where systemic anticoagulation was contraindicated, demonstrating its safety and efficacy.^[5,9]^ In our case, the absence of anticoagulation did not result in thrombotic complications, and the patient’s oxygenation remained stable throughout the procedure. This suggests that heparin-free ECMO is a viable option for patients undergoing high-risk airway surgeries, offering a balance between maintaining oxygenation and minimizing bleeding risks.^[3]^

In our case, where ECMO was run for 6 hours, no thrombotic events occurred, demonstrating the safety of short-term heparin-free ECMO. Previous literature indicates that the median safe time for ECMO without systemic anticoagulation ranges from 70 to 114 hours.^[18,19]^ Table 1 lists ECMO patients who were managed without heparin, and studies have found that oxygenators or circuit often need to be replaced approximately every 10 days. This case report adds to the growing body of evidence supporting the use of heparin-free ECMO, particularly in patients at high-risk of bleeding or those who develop heparin-induced thrombocytopenia. Despite the absence of systemic anticoagulation, the ECMO circuit and oxygenator remain at risk of thrombosis. High blood flow rates, antithrombotic-coated circuits, and frequent circuit and oxygenator changes are crucial to prevent this.^[6,7]^ Additionally, regular monitoring of coagulation markers (activated clotting time, activated partial thromboplastin clotting time, fibrin/fibrinogen degradation products, soluble fibrin, thrombin-antithrombin complex, plasmin-α2-plasmin inhibitor complex) and viscoelastic hemostatic assays such as TEG6s® is essential to assess coagulofibrinolytic status and guide blood product transfusion.^[8]^ Overall, the evidence suggests that heparin-free ECMO can be a safe and effective option for a wide range of patients, offering potential benefits in terms of reduced bleeding and improved outcomes. However, careful patient selection, monitoring, and management are crucial to mitigate the risks of thrombosis and bleeding. As more cases like this are reported, the evidence base will strengthen, potentially leading to revised guidelines that better balance bleeding and thrombotic risks in ECMO-treated patients.

This study is based on a single case, which limits the generalizability of the findings. The unique clinical features of the patient, including tumor location and the use of heparin-free ECMO, may not apply to all cases of airway obstruction. Additionally, the short ECMO duration (6 hours) and lack of long-term follow-up limit our ability to assess long-term safety and efficacy. The absence of anticoagulation also presents risks, highlighting the need for careful patient selection and monitoring.

## 5. Conclusion

This case report demonstrates the successful use of heparin-free VV-ECMO in managing severe airway obstruction caused by recurrent synovial sarcoma. The preemptive VV-ECMO maintained oxygenation and facilitated safe tumor resection, minimizing intraoperative bleeding risks. This highlights ECMO’s critical role in life-threatening airway obstructions and supports the use of heparin-free ECMO to balance effective respiratory support with reduced bleeding complications in high-risk surgeries.

## Author contributions

**Conceptualization:** Bin Sun, Meiyan Zhou.

**Data curation:** Bin Sun, Meiyan Zhou, Rongguo Wang, Qian Liu, Liwei Wang.

**Formal analysis:** Bin Sun, Meiyan Zhou, Rongguo Wang.

**Funding acquisition:** Bin Sun, Liwei Wang.

**Investigation:** Bin Sun, Meiyan Zhou, Jinghao Zhang.

**Methodology:** Bin Sun, Yan Zhang, Jinghao Zhang.

**Resources:** Bin Sun, Li Yan.

**Supervision:** Jinghao Zhang.

**Validation:** Bin Sun, Jinghao Zhang.

**Visualization:** Bin Sun, Rongguo Wang.

**Writing – original draft:** Bin Sun, Meiyan Zhou, Liwei Wang.

**Writing – review & editing:** Bin Sun, Rongguo Wang, Jinghao Zhang, Liwei Wang.
